# Power and variety-seeking: A compensatory perspective

**DOI:** 10.3389/fpsyg.2022.928958

**Published:** 2022-09-07

**Authors:** Jin Wang, Fei Jin

**Affiliations:** ^1^College of Literature and Journalism of Sichuan University, Chengdu, Sichuan, China; ^2^Department of Marketing, Sichuan University, Chengdu, Sichuan, China

**Keywords:** power state, variety-seeking, need for control, consumer knowledge, choice

## Abstract

In the current research, we show that low-power state promotes variety-seeking. We argue that this effect occurs because consumers in low-power state desire control and variety-seeking as a way to restore sense of control. The effect of power on variety-seeking is reduced when consumer knowledge in a certain consumption domain is high (vs. low) because knowledge is an alternative way to retain sense of control. Three experiments provide systematic evidence for this effect across different product categories. These findings contribute to the literature on how variety-seeking can be used as a way to compensate and enhance our understanding of power and consumer knowledge.

## Introduction

Variety plays a critical role in consumer choice, and companies often stimulate purchase by constructing an assortment. Consumers are easily attracted by varied product assortments (Broniarczyk et al., 1998; Hoch et al., 1999) and tend to choose varied choices rather than non-varied ones, even when varied choices contain fewer items they like (Ratner et al., 1999; Ariely and Levav, 2000). Previous research examines factors influencing consumers’ variety-seeking tendency from multiple perspectives, such as personality traits (Berlyne, 1970; Ariely and Levav, 2000), product characteristics (Gourville and Soman, 2005), and environmental factors (Levav and Zhu, 2009). However, how consumers’ internal situational states affect their choices related to variety remains an open question worthy of further exploration.

Contributing new insights to existing work on the topic, the current research tackles this question by examining how power states, one of the key psychological states of consumers, and impact variety-seeking. We propose that low-power state promotes consumers’ variety-seeking tendency which is rooted in the compensatory value of variety-seeking. Low-power state makes consumers feel a low sense of control, and seeking variety could meet the need to restore sense of control. However, if consumers have an alternative means to increase their power state, they will not seek variety. In this research, we explore the moderating role of consumer knowledge.

Our research extends the literature on power states, variety-seeking, and consumer knowledge in several ways. First, we enrich the related research on compensatory consumption (Rucker and Galinsky, 2008). Previous literature on compensation consumption has focused mainly on symbolic or status products (Rucker and Galinsky, 2008; Rucker et al., 2014), but our research demonstrates that consumers also perform strategic compensation for ordinary products through variety-seeking. Second, we contribute to the power states literature by showing that a lower-power state promotes variety-seeking behavior. Third, we demonstrate that consumer knowledge moderates the effect of power state on variety-seeking, providing a more nuanced understanding of how power state affects variety-seeking and how consumer knowledge influences decision making.

## Theoretical development

### Power states and need for control

As an important basis of social hierarchy, power refers to asymmetrical control over valuable resources (Magee and Galinsky, 2008) which can affect consumers’ thinking, feeling, perception, and behavior (Guinote, 2017). In consumer behavior research, power represents power states, i.e., consumers’ perception of power at the individual level (Magee, 2019). Power is either an individual’s ability to be independent from others or external influences, or, in an organizational structure, the perception of power related to social status in the long-term economic situation and position (Lee and Tiedens, 2001). In line with prior work, in our research we consider power state to be a relative psychological state in interrelationships that can be triggered by situational factors, roles, or memories of past experiences (Galinsky et al., 2003; Magee et al., 2007).

Low power refers to an aversive state (Keltner et al., 2003). People in low-power states often feel out of control in relation to their own or others’ behaviors (Rucker and Galinsky, 2008; Guinote and Lammers, 2016). Previous research suggests that loss of control leads to many negative consequences. For instance, low-power states enhance people’s sense of uncertainty, make them perceive fewer reward opportunities, and become even more vulnerable to external attack (Anderson and Galinsky, 2006; Brinol et al., 2007). Therefore, when people are in low-power states, they are motivated to change this aversive condition.

Consumption is one of the important approaches to improve this state. For example, low-power states promote consumers to purchase status-related products to compensate for lack of power, to prefer status symbolic advertisements and famous brands (Rucker and Galinsky, 2008), to purchase conspicuous products (Rucker and Galinsky, 2008), and prefer large size food/beverage (Dubois et al., 2012). These previous studies have focused on compensatory consumption from the perspective of the symbolic meaning of products. Although symbolic products can greatly make up for consumers’ lack of sense of control, we believe that consumers in low-power states can also compensate for a lack of control by choosing varied choices among ordinary products.

### Variety-seeking as a compensation behavior

Variety-seeking refers to a tendency to choose a variety in products or services (Ratner and Kahn, 2002); such variety indicates that items are distinct or differentiated from one another (Kahn and Wansink, 2004). Consumers choose a variety for many purposes, the most important of which is utility maximization. If variety can satisfy consumers’ need for utility, they will be more likely to choose variety (Inesi et al., 2011). For example, a consumer would be more likely to choose a considerable amount of variety if he or she believed that more chocolate flavors could bring greater utility. In our study, we focus on contexts in which consumers are faced with many choices but the utility of each choice is basically the same.

Making choices has long been considered a source of the sense of personal control (Langer, 1975). Previous studies have shown that offering multiple choices can enhance consumers’ sense of autonomy and happiness, and even the mere exercise of making choices can improve consumers’ sense of control (Iyengar and Lepper, 1999). Similarly, the behavior of seeking variety can make consumers think that they are independent and autonomous (Kim and Drolet, 2003), and such autonomy serves as an important component of sense of control. Therefore, we argue that if consumers have the motivation to fulfill a need for control, they will choose variety. Our hypothesis is supported to an extent by Inesi et al. (2011), who have shown that when consumers are deprived of control, they prefer a large choice set to small one, as a large choice set (vs. a small choice set) contains more various items. We therefore propose the following hypotheses,

*H1:*Low-power state promotes variety-seeking.

*H2:* Need for control mediates the effect of low-power state on variety-seeking.

### Consumer knowledge as a moderator

Consumer knowledge refers to consumers’ cognition or memory of products or experiences in a certain domain, including both subjective and objective knowledge. In this research, we focus on the subjective knowledge of consumers—i.e., the extent to which consumers believe they know about consumption or products in a certain domain (Sujan, 1985). Consumer knowledge plays a crucial role in information searching (Brucks, 1985; Rao and Sieben, 1992) and processing (Johnson and Russo, 1984). Prior work has investigated the effect of country-of-origin information on different consumers (low consumer knowledge vs. high consumer knowledge), and finds that consumers with low knowledge use country-of-origin information unilaterally to evaluate products, while consumers with rich consumer knowledge consider this information only when the product’s attribute information is relatively vague (Maheswaran, 1994). Cowley and Janus (2004) demonstrate that when consumer knowledge increases, consumers are less likely to be influenced by advertising.

With an increase of consumer knowledge, consumers can better appreciate products or services, distinguish between different features, and identify new features. For example, a consumer with rich knowledge of red wine can not only discern sweetness, acidity, tannin, fruit aroma, and wine body, but also distinguish subtle differences in degree of acidity. The related question we ask in this research is: How does consumer knowledge affect a variety of choices? Consumers with high consumer knowledge already know a certain domain in an extensive way, so they can confidently identify differences among various choices and can select the products they like from the choice set. Thus, a variety of choices fail to provide enough information for consumers with high consumption knowledge (vs. consumers with low consumption knowledge). Instead, these consumers are more likely to choose less variety and increase in-depth cognition of products (Clarkson et al., 2013). In contrast, for consumers with low consumer knowledge, choosing variety can provide as many opportunities as possible to expand their knowledge and enhance their ability to distinguish between different items (Tse and Wilton, 1988).

For consumers in low-power state, knowledge, as a source of power states (French et al., 1959), can compensate for a lack of sense of control. When choosing knowledge as an alternative means to enhance sense of control, they would therefore be less likely to choose variety. In contrast, consumers in high-power state make decisions based more on their own preferences than on their level of consumer knowledge; hence, the level of knowledge will not affect their variety-seeking tendency. Those with high knowledge will not seek variety. We therefore propose the following hypothesis:

*H3:* Consumer knowledge moderates the effect of power state on variety-seeking, such that when consumer knowledge is high (vs. low), consumers in low-power state show a lower variety-seeking tendency.

### Overview of studies

Four studies, including secondary data and experiments, examine how power states affect variety-seeking. Study 1, using online review data from Jingdong (jd.com), shows initial evidence for our hypothesis that there is a negative correlation between power state and variety-seeking. This evidence is important for proceeding to test the effect in real-life settings with high ecological validity. Based on this correlational study, we then conduct three experiments by manipulating participants’ power state in different ways and test the causal relationship between power state and variety-seeking using different products. Drawing on previous studies (Ratner et al., 1999; Etkin and Sela, 2016), we measure variety by counting the number of different items in a choice set. Specifically, Study 2 shows that a low-power state promotes consumers’ variety-seeking by manipulating power state through role-play. Study 3 provides convergent evidence of the effect by episodic priming and tests the mediating role of need for control. Study 4 examines the moderating role of consumer knowledge; i.e., it attenuates the effect for consumers in a low-power states. Our three experiments all adopt the same data screening criteria: whether participants pass an attention check (if not, their data are deleted.) and whether participants finish the writing task for the power manipulation as required (for detailed information, see Table 1). We adopted the same data screening criteria: whether participants pass an attention check (if not, their data are deleted.) and whether participants finish the writing task for the power manipulation as required (for detailed information, see Table 1).

**Table 1:** Study overview.

	***N***	**DV measure**	**IV measure**	**Mediator**	**Moderator**	**Samples**
**Study 1**	1,702	Variety-seeking (product items)	Membership level Magee and Galinsky, 2008			Secondary data from Jingdong
**Study 2**	98	Variety-seeking (shopping decision task)	Power state (low vs. high) Garbinsky et al., 2014			Experiment with Chinese online platform participants (student sample)
**Study 3**	176	Variety-seeking (shopping decision task)	Power state (episodic priming: low vs. high) Galinsky et al., 2003	Need for control		Experiment with Mturk participants (adult sample)
**Study 4**	120	Variety-seeking (as in Study 3)	Power state (as in Study 2)		Consumer knowledge Clarkson et al., 2013	Experiment with Chinese online platform participants (student sample)

## Study 1: Pilot study

The purpose of the pilot study was to examine whether power state and variety-seeking were correlated. We analyzed transaction data from jd.com, a popular online ecommerce platform in China. Previous studies have demonstrated that a person’s power state is affected by his or her status (Magee and Galinsky, 2008). In our study, we aimed to examine whether consumers’ level of membership was negatively correlated with variety-seeking. Membership-level is set up by Jd.com for its users through a specific membership grading system. Consumers in each level embody corresponding privilege of Jingdong Mall, such as free shipping, special price, VIP gift, etc. Generally, membership-level depends on growth value, which is calculated by usual login, shopping history, number of shopping days, as well as comments data. The membership grading system will automatically provide relevant grades to the users, with no additional licensing efforts. In addition, membership levels are not necessarily associated with economic situations. Consumers who buy a lot of cheap products from this platform can have a higher membership level than those who buy an expensive product. Data containing ten products from jd.com were captured through R software in late January 2018. The reason we used the data from jd.com is that data on this platform included relatively complete information on consumers’ level of membership, and jd.com itself executes a specific membership grading system, an ideal proxy variable for power state.

### Procedure

Using the data that contained information about the products that consumers chose, we captured a total of 1,702 reviews for five products (Cookies, Nuts, Chips, Candy, and Yogurt). As variety is the key variable in our study, the products themselves had to have multiple options. There were no significant price differences among these products.

#### Variety-seeking

**Figure 1:**
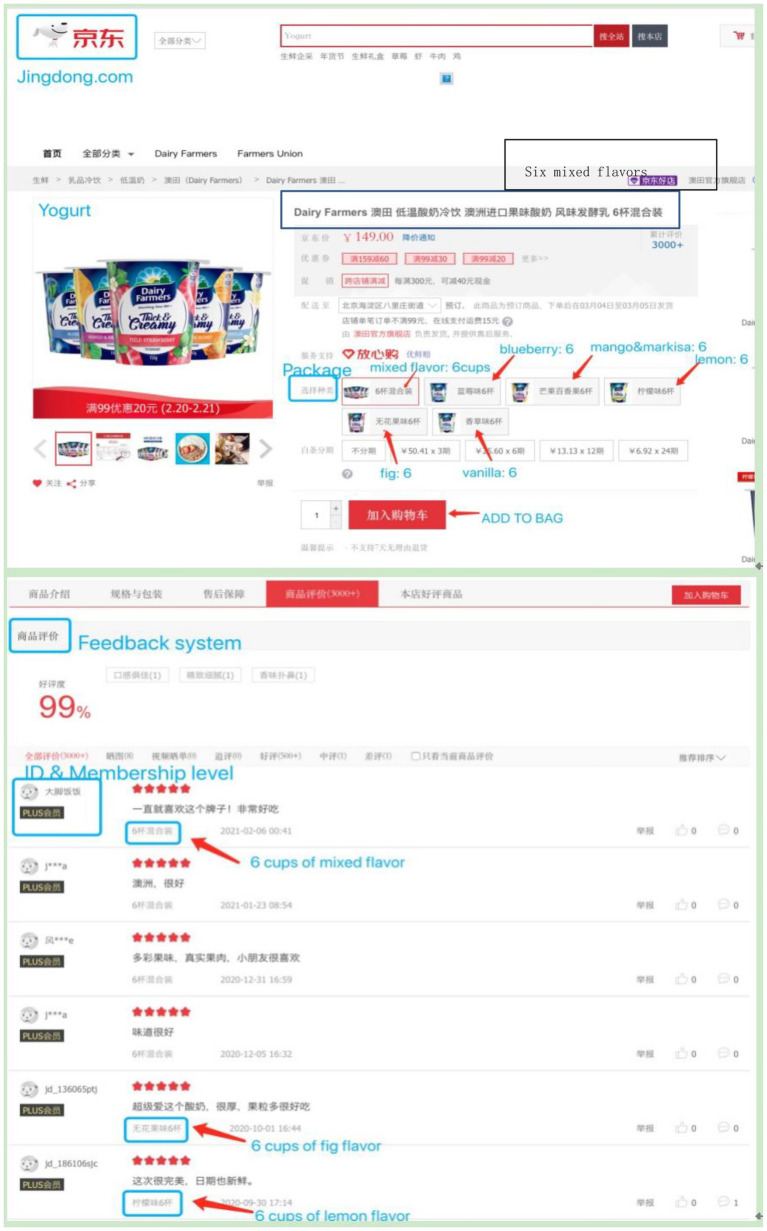
Screenshot of product details on Jingdong: Take Yogurt as example (https://item.jd.com/64480252426.html).

We coded variety-seeking in accordance with sellers’ presentation of product items. If a consumer chose varied items, we coded 1; otherwise, we coded 0. Take yogurt as an example: if a consumer chose a packet of mixed flavors (e.g., blueberry, strawberry, orange, and mango), we coded 1; if a consumer chose a packet of a single flavor (e.g., mango), we coded 0 (Figure 1).

### Results and discussion

We predicted that consumers with a higher membership level would be less likely to seek variety, suggesting a negative correlation between membership level and variety-seeking. To test this hypothesis, membership level, from registered member to diamond VIP member, was coded between 1 and 5 (1, lowest membership level; 5, highest membership level). Results indicated that membership level was negatively correlated with variety-seeking [*r* (1702) = −0.34, *p* < 0.001, *Fisher’s Z* = 0.35].

In Study 1, the negative correlation between consumer membership level and variety-seeking was shown by secondary data from jd.com, which increased the ecological validity of our research. However, from a correlational analysis, we could not claim there was a causal relation between power state and variety-seeking. There might be a number of alternative explanations for this correlation. For example, high membership level may represent an experienced consumer for whom a tendency toward variety-seeking might be reduced, or it may represent consumers with a strong internal tendency toward variety-seeking. Therefore, we conducted three experiments to test for a causal relationship between power state and variety-seeking by manipulating power states directly.

## Study 2: Power states and variety-seeking

In this study, we manipulated participants’ power state to find direct evidence that consumers in low-power state (vs. high-power state) were more likely to seek variety.

### Procedure

A total of 100 students from a Chinese online platform participated in this study, but 2 participants were ruled out due to failing the attention check. The remaining 98 participants (51.02% females, *M*_age_ = 23.06, *SD* = 2.67) were randomly assigned to two groups (low-power state vs. high-power state). They were told that the study was about role play and shopping behavior and they would be paid 3 RMB for completing two unrelated tasks.

#### Power manipulation

The manipulation method was adapted from Garbinsky et al. (2014). Because the participants were university students, we tailored the power manipulation context to education. Specifically, participants in the high-power (vs. low-power) condition were told that they had been assigned a group project worth 75% of their final grade, and the professor had selected them to be group leader (vs. member). Each team had 10 group members who would listen to the leader’s instructions. At the end of this class, the leader would evaluate group members’ performances, which would be part of the project grade, but members had no opportunity to evaluate the group leader.

#### Variety-seeking

Once participants had completed the power manipulation, they were guided to the second part of the study. We asked participants to imagine that they were buying socks and had found a desired shop. The seller was offering discounts and consumers could choose five pairs of socks from nine different colors. Consumers could choose five pairs of socks with the same color or different colors, but the total number must be five. We measured variety-seeking by counting the number of different colors participants chose.

Previous research about the experimental materials used to study variety-seeking has focused mostly on hedonic products (such as candies, drinks, songs, etc.), because consumers tend to show a higher variety-seeking tendency toward these kinds of products (vs. utilitarian products) (Ratner and Kahn, 2002; Kahn and Ratner, 2005). In order to make our study more rigorous, we conducted a pretest involving 32 university students (40.63% female, *M*_age_ = 20.06, *SD* = 1.81) from the same sample pool (Drolet et al., 2007). Participants were asked to rate two items (presented in random order): “Your decision to choose which pair of socks to buy will be mainly based on functional facts” and “Your decision to choose which pair of socks to buy will be based a lot on feeling,” using a seven-point scale (1, strongly disagree; 7, strongly agree). By comparing ratings with the scale midpoint of 4, the results of the single-sample t-test showed that the average utilitarian score was significantly higher than 4 [*M* = 4.94, *SD* = 1.46, *t*(31) = 3.64, *p* = 0.001, Cohen’s *d* = 1.29], while the average hedonic score was significantly lower than 4 [*M* = 3.09, *SD* = 1.75, *t*(31) = −2.93, *p* = 0.006, Cohen’s *d* = 1.04].

Then, we asked participants how appealing the socks were and how much they liked them (1, not at all; 7, very much). Finally, we collected standard demographic information.

### Results and discussion

#### Manipulation check

We compared the two power conditions (high-power vs. low-power) by asking “How powerful did you feel in the group?” The results of a one-way ANOVA indicated that participants in the high-power group felt more powerful than those in the low-power group (*M*_high-power state_ = 6.06, *SD* = 0.72; *M*_low-power state_ = 4.16, *SD* = 94.65, *p* < 0.001, Cohen’s *d* = 2.78). Meanwhile, there was no significant difference between the two groups in positive emotions [*M*_high-power state_ = 5.27, *SD* = 1.24, *M*_low-power state_ = 4.98, *SD* = 1.11, *F*(1, 96) = 1.45, *p* = 0.23, *ns*], or negative emotions [*M*_high-power state_ = 3.08, *SD* = 1.38, *M*_low-power state_ = 3.39, *SD* = 1.32, *F*(1, 96) = 1.26, *p* = 0.27, *ns*], indicating that our manipulation of power state was successful and did not affect participants’ emotions.

#### Variety-seeking

A general linear model analysis was performed with power state (high-power state = 2, low-power state = 1) as the independent variable and variety-seeking as the dependent variable. Results demonstrated that the main effect of power state was significant [*F*(1, 96) = 7.21, *p* = 0.009, η^2^ = 0.07], and participants in the low-power state were more likely to choose variety than those in the high-power state (*M*_low-power state_ = 4.33, *SD* = 0.99 vs. *M*_high-power state_ = 3.61, *SD* = 1.58). Participants from the two groups showed no difference in how much the socks appealed to them (*p* = 0.81, *ns*), or in how much they liked the socks (*p* = 0.78, *ns*).

Consistent with our hypothesis, the results of Study 2 support the hypothesis that consumers in low-power state are more likely to seek variety.

## Study 3: Mediating role of need for control

Study 3 had two goals. First, we changed the experimental context to provide further evidence for our main effect. Second, we probed the underlying mechanism. We proposed that consumers in low-power state seek variety to satisfy their need for control. We also ruled out an alternative explanation of self-expression, as previous research suggests that variety-seeking can meet people’s self-expression needs (Kim and Drolet, 2003; Fernandes and Mandel, 2014).

### Procedure

A total of 180 participants from MTurk participated in this study, but 4 were not included in the data analysis due to failing the attention check. The final 176 participants (37.50% females, *M*_age_ = 34.07, *SD* = 9.22) were randomly assigned to two groups (low-power state vs. high-power state). They were paid $0.50 to complete two unrelated tasks.

#### Power manipulation

Participants were first asked to complete an episodic priming manipulation of power. Specifically, participants were instructed to write about an event in which they felt powerless/an event in which they felt powerful/an ordinary event that happened the day before (Galinsky et al., 2003). Previous studies have shown that this recall task has good reliability and validity in eliciting power states (Anderson and Galinsky, 2006; Rucker and Galinsky, 2008).

#### Variety-seeking

After the power state manipulation, participants were asked to complete a shopping decision task: when you are browsing the website, you find a chocolate seller offering the promotion “choose whatever you like.” Consumers can buy six chocolates, of one flavor or different flavors. In order to rule out the influence of the existing brand on choices, the brand was not mentioned.

After making decisions, participants were asked to answer questions related to the two constructs--need for control and self-expression, and they were presented in a random order.

#### Need for control

Drawing on previous studies (adapted from Burger and Cooper, 1979; Consiglio et al., 2018), three items were used to measure participants’ need for control: “When I was making these decisions, I hoped to be able to control what I could do at a certain time”; “When I was making these decisions, I wanted to have control”; “I want to be in control most of the time in my daily life.” (1, not at all; 7, very much; *α* = 0.94).

#### Self-expression

With reference to previous studies (Fernandes and Mandel, 2014), four items were adopted to measure participants’ self-expression: “The chocolate I chose provides others enough information about me”; “When I was making decisions, I considered whether the chocolate could express myself”; “The chocolate I chose shows that I have unique taste”; “The chocolate I chose shows a lot about what kind of person I am.” (1, not at all; 7, very much; *α* = 0.74). Finally, we solicited standard demographic information.

### Results and discussion

#### Manipulation check

Two research assistants (RAs) majoring in English rated the participants’ recall tasks (“To what extent, did the participants’ recall task reflect his/her power states?” 1, not at all; 7, very much). They were told the definition of power, and were instructed to go through all of the participants’ descriptions before rating. To make sure that the RAs understood the definition of power and the main points for rating, 10 samples were randomly extracted for them to practice on. Finally, they separately rated all of the participants’ descriptions and consistent ratio of 90.66% with disagreement solved.

A one-way ANOVA was performed on the average of the two RAs’ scores. The results showed that the power state of participants in the high-power group was significantly higher than that of participants in the low-power group [*M*_high-power state_ = 4.61, *SD* = 1.60; *M*_low-power state_ = 2.29, *SD* = 0.57; *F*(1, 174) = 164.19, *p* < 0.001, Cohen’s *d* = 2.73].

#### Variety-seeking

A general linear model analysis was performed with power state (high-power state = 2, low-power state = 1) as the independent variable and variety-seeking as the dependent variable. The results indicated that the main effect of power state was significant and participants in low-power state were more likely to choose variety than those in high-power state [*M*_low-power state_ = 4.23, *SD* = 1.40; *M*_high-power state_ = 3.56, *SD* = 1.52; *F*(1, 174) = 9.27, *p* = 0.003, η^2^ = 0.05].

#### Mediation effect

**Figure 2:**
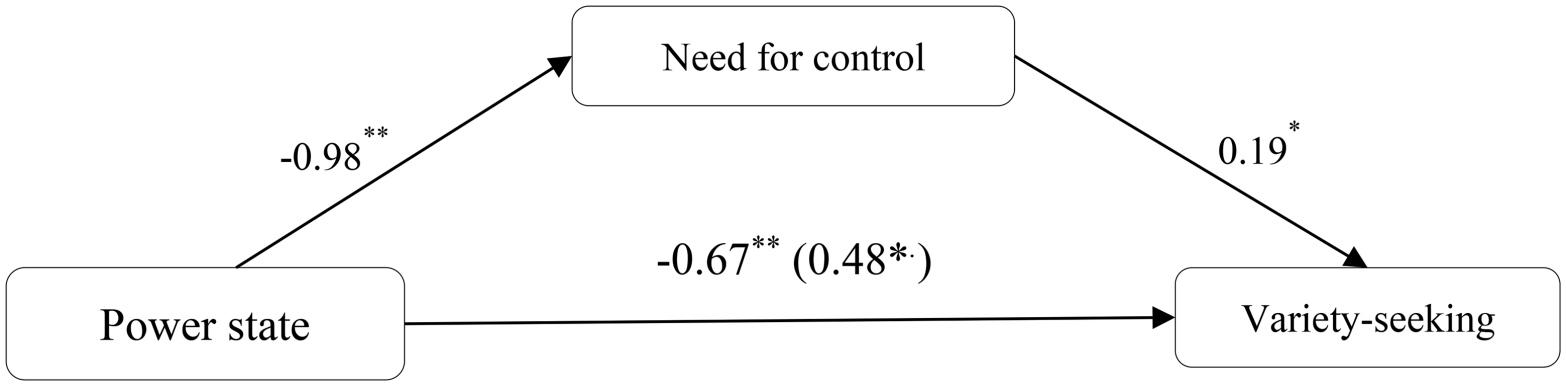
Study 3: Mediating role of need for control. ^*^, ^**^ and ^***^ represent <0.05, <0.01, and 0.001% significance, respectively.

First, a mediating effect analysis was conducted with power state as the independent variable, need for control as the mediator, and variety-seeking as the dependent variable (Preacher et al., 2007; SPSS Process Macro Model 4, *N* = 5,000). The results demonstrated that the effect of power state on need for control was significant [*B* = −0.98, *t*(174) = −4.74, *p* < 0.001]. A regression analysis with power state and need for control showed a significant effect of need for control on variety-seeking [*B* = 0.19, *t*(173) = 2.45, *p* = 0.02]. The effect of power state on variety-seeking remained significant, but the significance decreased [*B* = −0.48, *t*(173) = −2.08, *p* = 0.04]. Furthermore, the indirect effect of need for control was significant [95% CI = (−0.40, −0.03)], indicating that the mediating effect of need for control was significant (see Figure 2).

To test whether self-expression is an alternative explanation, the mediating effect of self-expression was examined in the same way. Results show that the indirect effect of self-expression included 0 and was not significant [95% CI = (−0.16, 0.02)]. Therefore, the explanation that people in low-power state seek variety for self-expression is ruled out in this study.

In Study 3, when we changed the manipulation of power state and the stimulus of variety-seeking, the hypothesis that consumers in low-power state (vs. high-power state) are more likely to seek variety was supported. More importantly, we verified the mediating effect of need for control and ruled out the role of self-expression.

## Study 4: Moderating role of consumer knowledge

The studies above demonstrate that low-power state increases consumers’ tendency to seek variety, and they use variety-seeking as a way to satisfy their need for control. We were also interested in the possibility of a boundary condition for the positive relationship between low-power state and variety-seeking. Do all consumers seek variety to satisfy their need for control when experiencing low-power state? In Study 4, we answered this question by exploring the moderating role of consumer knowledge.

### Procedure

A total of 120 university students (57.50% female, *M*_age_ = 22.56, *SD* = 2.28) from a Chinese online platform were paid $2 to participate in this study. They were randomly assigned to two groups (low power state vs. high power state), with consumer knowledge as a continuous variable. Participants were told they would complete two unrelated studies: the first about daily life role-playing, and the second about chocolate preference.

#### Power state manipulation

As in Study 2, a role-play was used to manipulate participants’ power state.

#### Variety-seeking

The scenarios and measurement of variety-seeking were consistent with those in Study 3.

#### Consumer knowledge

In this study, we focused on consumers’ subjective knowledge, i.e., their own cognition and judgment of consumption knowledge in a certain domain. With reference to previous studies (Clarkson et al., 2013), two items were designed to measure consumer knowledge: “In general, how much do you know about chocolate?”; “In general, what is your knowledge about chocolate?” (1, not at all; 7, very much; *α* = 0.83).

After making decisions, participants were asked how much they liked chocolates and how much they thought the chocolates in the experiment were appealing (1, not at all; 7, very much). Finally, we solicited standard demographic information.

### Results and discussion

#### Manipulation check

The results of one-way ANOVA indicated that the power state of participants in the high-power state group was significantly higher than that of those in the low-power state group [*M*_high-power state_ = 4.97, *SD* = 1.53; *M*_low-power state_ = 3.07, *SD* = 1.42; *F*(1, 118) = 49.60, *p* < 0.001, Cohen’s *d* = 1.82], revealing that our manipulation of power state was successful.

#### Variety-seeking

We conducted a regression analysis with variety-seeking as the dependent variable, and power state (high-power state = 1, low-power state = −1), mean-centered consumer knowledge, and their interaction as independent variables. The results indicated that the interaction of power state and consumer knowledge was significant (*B* = 0.20, *t* = 2.41, *p* = 0.02, Cohen’s *d* = 0.41), the main effect of consumer knowledge was significant (*B* = −0.33, *t* = −3.87, *p* < 0.001, Cohen’s *d* = 0.70), but the main effect of power state was not significant (*p* > 0.1).

**Figure 3:**
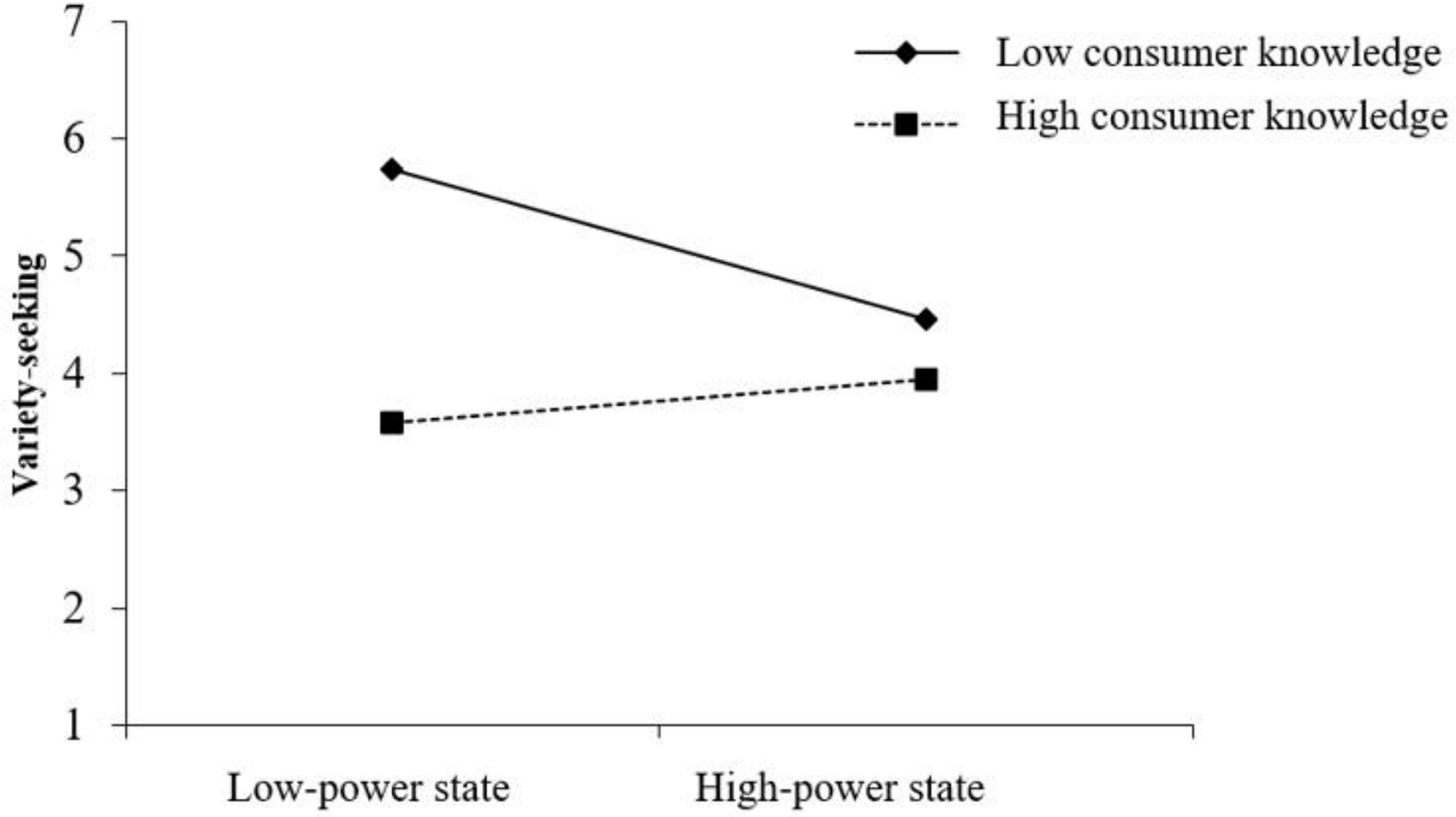
Study 4: Moderating role of consumer knowledge.

To further identify the moderating effect, we conducted a spotlight analysis. The spotlight analysis of consumer knowledge (±1 *SD*) (Irwin and McClelland, 2001; Spiller et al., 2013) illustrated that in the condition of low consumer knowledge (vs. high consumer knowledge), consumers in low-power state showed a higher variety-seeking tendency [*B* = −1.04, *t* = −4.53, *p* < 0.001, (−1.50, −0.59)], but consumer knowledge did not affect the variety-seeking tendency of participants in high-power state [*B* = −0.23, *t* = −0.04, *p* = 0.37, (−0.72, 0.27), *ns*]. Furthermore, in the condition of low consumer knowledge, participants in low-power state showed higher variety-seeking tendency than those in high-power state [*B* = −0.57, *t* = −2.875, *p* = 0.01, (−0.97, −0.17)]. However, in the condition of high consumer knowledge, there was no significant difference between consumers in high or low-power states in variety-seeking tendency [*B* = 0.11, *t* = 0.56, *p* = 0.58, (−0.28, 0.51), *ns*] (see Figure 3).

Study 4 supports the interaction effect of consumer knowledge and power state on variety-seeking; i.e., consumers in low-power state who possess less consumer knowledge were more likely to seek variety.

## General discussion

Variety-seeking is an important characteristic in contemporary society and it is influenced by numerous factors. The current research explores whether, why, and when power state affects variety-seeking. Four studies provide convergent evidence that variety-seeking can be used as a way to compensate for lacking sense of control. Results from Study 1, using secondary data from jd.com, provide support for the negative correlational relationship between power state and variety-seeking. Study 2 then demonstrates a causal relationship between power state and variety-seeking and shows that low-power state increase variety-seeking. Study 3 shows further support and demonstrates the underlying mechanism of need for control. Study 4 provides more evidence for our proposed effect by showing that consumer knowledge moderates the relationship between power state and variety-seeking; i.e., high consumer knowledge reduces the variety-seeking tendency of consumers in low-power state.

### Theoretical contributions

Our research provides several theoretical contributions. First, our findings shed light on compensatory consumption. Traditional compensatory consumption paradigms concentrate mainly on symbolic products, such as luxury goods and status-related products (Rucker and Galinsky, 2008), as these kinds of products represent consumers’ social status (one of the sources of power states). However, none of the products in our research have obvious symbolic characteristics. Consumers in a low-power state (vs. high-power state) had a higher variety-seeking tendency, indicating that variety-seeking can also be a way to compensate (Inesi et al., 2011). Our findings illustrate that for consumers in low-power state, need for control can be satisfied through variety-seeking rather than symbolic self-completion.

Second, this research furthers understanding of power states on consumer behavior. In a review, Guinote (2017) points out that, as an important psychological state of individuals, the impact of power states on consumers’ cognitive judgment is worth exploring in many aspects. The current research examines the impact of power states on variety-seeking (one of the most important aspects of consumer behavior), which has not been directly discussed in previous studies.

Third, our research extends understanding of the impact of consumer knowledge on purchase decisions. Study 4 reveals that consumers in a low-power state show a higher variety-seeking tendency when they have low consumer knowledge (vs. high consumer knowledge). This finding indicates that variety-seeking is actually a selective strategy, and consumers’ own knowledge in a certain domain can change the desirability of variety-seeking. Clarkson et al. (2013) discover that when facing a new consumption experience, people with less consumer knowledge tend to choose varied experiences to obtain more utility, while those with more consumer knowledge tend to choose less experience to deepen their understanding of a certain domain. However, our research finds that consumer knowledge can affect the need for a variety of consumers in a low-power state, influenced not by maximizing utility but by satisfying the motivation to restore a sense of control. These findings complement research on how consumer knowledge affects variety and consumers’ decision-making.

Fourth, our research enhances understanding of variety. Previous studies explore factors influencing variety-seeking mainly from the perspectives of social factors and self-presentation (Ariely and Levav, 2000; Ratner and Kahn, 2002; Etkin, 2016). Less attention has been paid from the perspective of consumers’ psychological factors, especially on incidental ones. In our work, the primed power states are not related to consumption scenarios, but they still significantly impact variety-seeking. A prior study argues that variety-seeking is unlikely to occur for consumers with a high level of perceived risk (Hoyer and Ridgway, 1984). In an ancillary study, we manipulate product price as a measure of perceived risk, to test whether our proposed effect would be affected when people face different product values. The results show that a main effect of power on variety-seeking still occurs, thus providing further evidence for the relationship between power and variety-seeking (see Appendix).

### Managerial implications

Our results also have managerial implications for businesses and marketing managers. First, in market segmentation, companies can consider the strategy of increasing the variety of products if consumers have the characteristics of low-power states (such as low income and low social status). Companies can enhance consumer knowledge of target groups if they do not want consumers to switch to competitors due to variety-seeking.

Second, although many sellers always emphasize variety in their promotion activities, our results suggest that such advertising strategies may not be effective for consumers with rich knowledge in a certain domain. For those in high-power state and with rich consumer knowledge, a single and deep strategy is better than one of variety.

### Limitations and future research directions

A few potential limitations merit discussion and provide some implications for future research. First, we did not differentiate the product types of variety-seeking in our experiments. In real life, however, products have many aspects, such as brand, flavor, product line, and so on. Does variety-seeking within different brands or different product category differ? Further study could explore different consumption contexts to refine understanding of variety-seeking.

Second, post-purchase satisfaction following variety-seeking is worth further exploration. Our research focuses on how power state affects variety-seeking, but this is only the first step in decision-making. What happens when consumers in low-power states complete their variety-seeking? Will they be more satisfied or less satisfied? Although most previous studies have shown that consumers are more attracted to varied choices (Berlyne, 1970; Faison, 1977), less research focuses on satisfaction after choosing variety. One exception is the study of Etkin and Sela (2016) in which they argue that various product usage experiences decrease people’s post-purchase satisfaction because the experience makes consumers worry that the product is not used with high frequency. Future research could give more evidence on this question.

Third, more boundary conditions could be explored in the future. For example, from the perspective of individual factors, how consumers perceive their current power state may moderate the relationship between power state and variety-seeking. Specifically, if consumers in a low-power state believe that this state is immutable (vs. changeable), they may not regard variety-seeking as a way to regain their sense of control, believing that the status quo is irreversible. From the product perspective, since purchasing a powerful brand can provide a way for consumers to regain their sense of control, do both powerful brands and weak brands affect consumers’ variety-seeking (Sundar and Noseworthy, 2014)? When faced with weak brands, perhaps consumers in a low-power state would be less likely to seek variety.

## Author’s note

The content of this manuscript has been presented in the competitive session at the Association for Consumer Research Conference (*Jin and Zhang, 2020).*

## Data availability statement

The raw data supporting the conclusions of this article will be made available by the authors, without undue reservation.

## Ethics statement

Ethical review and approval was not required for the study on human participants in accordance with the local legislation and institutional requirements. The patients/participants provided their written informed consent to participate in this study.

## Author contributions

All authors listed have made a substantial, direct, and intellectual contribution to the work and approved it for publication.

## Conflict of interest

The authors declare that the research was conducted in the absence of any commercial or financial relationships that could be construed as a potential conflict of interest.

## Publisher’s note

All claims expressed in this article are solely those of the authors and do not necessarily represent those of their affiliated organizations, or those of the publisher, the editors and the reviewers. Any product that may be evaluated in this article, or claim that may be made by its manufacturer, is not guaranteed or endorsed by the publisher.
